# Time to death and its predictors among adult patients with COVID-19: A retrospective cohort study in Ethiopia

**DOI:** 10.3389/fepid.2022.1065184

**Published:** 2023-01-17

**Authors:** Tegene Atamenta, Amsale Cherie, Wudma Alemu

**Affiliations:** ^1^ School of Nursing, College of Health Science, Woldia University, Woldia, Ethiopia; ^2^ School of Nursing and Midwifery, College of Health Science, Addis Ababa University, Addis Ababa, Ethiopia

**Keywords:** adult patients, COVID-19, Ethiopia, time to death, predictors

## Abstract

**Background:**

Coronavirus (COVID-19) disease affected people throughout the globe and has become a severe threat to the health and wellbeing of the global community. Time to death and predictors of mortality vary across settings. So far, no or few related studies have been undertaken in Ethiopia. Studying the time to death from COVID-19 and its predictors is essential to understand the characteristics of the disease and thereby contribute to the identification of indicators for early detection and initiation of treatment. Therefore, this study aimed to estimate time to death and its predictors among adults with COVID-19 in Ethiopia.

**Methods:**

A retrospective follow-up study was conducted among 602 adults with COVID-19 attending Eka Kotebe General Hospital, COVID-19 Treatment Center, between 13 March 2020 and 13 November 2020. The data were entered by Epi-data version 4.2 while the analysis was carried out using STATA version 16. A Kaplan–Meier survivor curve was computed to estimate the survival probabilities. A log-rank test was used to compare the difference in survival curves. Cox proportional hazard models were fitted to identify the predictors of time to death.

**Results:**

The overall median time to death was 21 days. Older adults (aged ≥65 years) [adjusted hazard ratio (AHR) 2.22, 95% confidence interval (CI) 1.02–4.86], being men (AHR 3.04, 95% CI 1.61–5.74), shortness of breathing at admission (AHR 2.29, 95% CI 1.16–4.54), comorbidity (AHR 2.23, 95% CI 1.04–4.80), diabetes mellitus (AHR 2.31, 95% CI 1.30–4.08), altered cardiac function (AHR 2.07, 95% CI 1.21–3.43), and baseline white blood cell count of greater than 10 (103/µl) (AHR 2.62, 95% CI 1.55–4.44) were independent predictors of COVID-19 mortality.

**Conclusion:**

Male sex, older adults, shortness of breathing at admission, patients with comorbidities, and higher blood cell count were significant predictors of time to death from COVID-19. Therefore, concerned stakeholders should focus on those predictors of mortality and design interventions accordingly to enhance the survival of patients with COVID-19.

## Introduction

The human coronavirus (COVID-19) is a large group of coronaviruses infecting multiple organs, mainly the respiratory system, ranging from the common cold to bronchiolitis and pneumonia. The coronavirus is named after its crown-like shape (1). The COVID-19 is a respiratory tract disease caused by a newly occurring coronavirus species called SARS-CoV-2. It was first identified in China, in Wuhan, Hubei Province, in December 2019. On 7 January 2020, in Wuhan, Chinese researchers started isolating a new individual infected by a coronavirus (2). The WHO announced on 11 March 2020 that the emerging COVID-19 disease was a global pandemic (3). In Ethiopia, the first case of COVID-19 was detected on 13 March 2020, in a 48-year-old Japanese man with a travel history. Within a few days, another three cases of COVID-19 were identified, who had had contact with the first case (4). To date, the country has tested more than five million individuals with suspected COVID-19, of which 494,180 cases were confirmed positive and, of these, 7,572 died, and 472,171 recovered (5).

Globally, the distribution and seriousness of the virus have increased dramatically (6). According to the latest WHO weekly epidemiological report, the virus appeared in more than 218 countries and territories, and over 624 million confirmed cases and over 6.5 million deaths have been reported globally. Furthermore, the highest number of deaths were reported in the United States (>1.1 million), India (>0.5 million), and France (>150,000) (7).

The likelihood of COVID-19 mortality rises with age. Studies showed that men had risk of death 11.5% higher than women. Renal disease, chronic lung diseases, and diabetes mellitus increased the risk of death by 52.3%, 89.5%, and 41.3%, respectively (8). The rates of spread of COVID-19 are higher than influenza and the likelihood of mortality is also greater. While most patients are estimated to have a remarkable improvement, the prognosis of older adults with the underlying disease may be poor (9).

The survival time varies across different countries. In the Democratic Republic of the Congo (DRC), Africa, the median survival from the first day of hospitalization among all admitted patient with COVID-19 is 9 days. The overall probability of survival at 2, 3, 5, and 10 days was 90%, 78.7%, 73.0%, and 71.6%, respectively (10). In Brazil, the median survival time from the first day of hospitalization was 12 days. The overall survival rate was 79.21% at 5 days and 59.22% at 10 days of hospitalization (11). In Mexico, the survival rate at 3 days was 86.9% (12), and in another set of Brazil numbers, the probability of survival at 10 days was 95.1% (13). A prospective observational cohort study among adult patients (aged ≥18 years) in New York found that the overall median survival time from the date of hospital admission was 9 days in New York City (14). A multicenter observational study in Mexico among critically ill patients reported a median survival time of 25 day from the first day of admission to the intensive care unit (15). Furthermore, a study from China among severely and critically ill patients found a median survival time of 25 day from the first confirmed positive result of a reverse transcription (RT)-PCR test (16).

Older age (14), male sex (17, 18), shortness of breath on admission (19, 20), chronic pulmonary diseases (21), diabetes (21, 22), cardiovascular diseases (23, 24), hypertension (25), severe acute kidney injury (21, 24), asthma (26), hypoalbuminemia (27), and increase in white blood cells (WBC) (28) were factors that decreased the survival status of patients with COVID-19.

The time to death from COVID-19 varies across different settings. Studying the time to death from COVID-19 and its predictors is essential to understand the characteristics of the diseases and thereby contribute to the identification of indicators for the early detection and initiation of treatment. So far, no or few related studies have been undertaken in Ethiopia. Little is known about the time to death from COVID-19 and its predictors, and even the process of the disease is not clearly known. The reports of time to death in different studies are also inconclusive. Therefore, the aim of the present study was to investigate time to death from COVID-19 and its predictors among adult patients in Ethiopia.

## Materials and methods

### Study design and setting

A retrospective follow-up study was conducted among 602 adult patients with COVID-19 confirmed by RT-PCT admitted to Eka Kotebe General Hospital, COVID-19 Treatment Center. Eka Kotebe General Hospital is the first COVID-19 treatment center in Ethiopia. The center is located in Addis Ababa, the capital of Ethiopia, where the highest number of cases of COVID-19 was recorded among all towns in Ethiopia. For the first time in Ethiopia, on 13 March 2020, 450 infected and suspected patients entered this facility, and 73 patients were confirmed to have COVID-19. The Eka Kotebe COVID-19 Treatment Center was relatively the only well-quipped treatment center in terms of ventilator machine, availability of the COVID-19 diagnosis test, and healthcare providers. According to a study in Ethiopia, the median time from the onset of symptoms to admission to hospital (diagnosis) was 4 days (interquartile range 3–5 days) for all cases.

### Study participants

The source population was adult patients (aged ≥18 years) with COVID-19 admitted to the Eka Kotebe General Hospital COVID-19 Treatment Center. The study population was adult patients with COVID-19 who attended the Eka Kotebe General Hospital COVID-19 Treatment Center between 13 March 2020 and 13 November 2020.

The inclusion criteria were as follows: (1) medical records of patients with confirmed COVID-19 at Eka Kotebe General Hospital COVID-19 Treatment Center; (2) patients aged 18 years and above; and (3) patients admitted during the study period.

The exclusion criteria were as follows: (1) incomplete patient charts (i.e., charts without the date of hospitalization and the date on which the outcome occurred, charts without final outcomes, such as death or censored); and (2) some independent variables.

### Variables

The dependent variable was time to death.

Independent variables were as follows: (1) sociodemographic factors, such as age and sex; (2) clinical characteristics on admission (cough, fever, shortness of breath, fatigue, myalgia, chest pain, sore throat, blood pressure, and oxygen saturation); (3) comorbidities [having an existing comorbidity, number of comorbidities, cardiac disease, congestive heart failure (CHF)], hypertension, diabetes mellitus, chronic lung diseases, HIV/AIDS, malignancy, chronic liver disease, renal diseases, and chronic neurological diseases); (4) treatment-related factors (azithromycin, dexamethasone, ceftriaxone, and vancomycin); and (5) laboratory findings (neutrophil, monocyte, lymphocyte, red blood count, hemoglobin hematocrit, platelet count, urea, creatinine, AST, ALT, random blood sugar (RBS), and blood group).

### Operational definitions

Time to death is the time interval from the first day of hospitalization to the time when the death of a patient is confirmed. The event of interest in this study was the occurrence of death due to COVID-19 at the time of hospital stay between 13 March 2020 and 13 November 2020. The follow-up time is the time from the first day of hospitalization until an event occurred. Censored indicated patients who did not develop the outcome of interest (death) during the follow-up period. The baseline laboratory marker was the first laboratory marker after hospitalization.

The severity of COVID-19 was classified as follows: (1) Asymptomatic infection: individuals who test positive for SARS-CoV-2 using a virologic test or an antigen test) but who have no symptoms that are consistent with COVID-19; (2) mild illness: individuals who have any of the various signs and symptoms of COVID-19 (e.g., fever, cough, sore throat, malaise, headache, muscle pain, nausea, vomiting, diarrhea, loss of taste and smell) but who do not have shortness of breath, dyspnea, or abnormal chest imaging; (3) moderate illness: individuals who show evidence of lower respiratory disease during the clinical assessment or imaging and who have an oxygen saturation measured by pulse oximetry (SpO_2_) ≥94% on room air at sea level; and (4) severe illness: individuals who have an SpO_2_ <94% on room air at sea level, a ratio of arterial partial pressure of oxygen to fraction of inspired oxygen (PaO_2_/FiO_2_) <300 mmHg, a respiratory rate >30 breaths/min, or lung infiltrates >50% (29, 30).

### Statistical analysis

Data entry was done by Epi-data version 4.2 and the analysis was carried out using STATA version 16. The incidence of death was calculated for the entire study period per 1000 person-days. A survival table was employed to estimate survival probabilities after diagnosis of COVID-19 at different time intervals. A Kaplan–Meier survivor curve was computed to estimate the survival time. A log-rank test was used to compare the difference in survival curves. A Cox proportional hazard regression model was fitted to identify significant predictors. The variance inflation factor (VIF) and tolerance were computed to check the existence of multicollinearity before running the multivariable Cox proportional hazard regression model. A VIF above 4 or tolerance below 0.25 indicated that multicollinearity might exist (31). In this study, the maximum VIF was 2.33 for comorbidity status with a mean VIF of 1.42 and the minimum tolerance value was 0.43. Thus, there was no multicollinearity between covariates (Supplementary File S1). Based on the global test result of Schoenfeld residuals, all the covariates fulfill the PH assumption (chi-square = 12.73and *p*-value = 0.6234). The crude hazard ratio (cHR) was computed in a bivariate analysis to see the strength association of a single variable with the outcome variable. Variables with a *p*-value of ≤0.25 in the bivariate analysis were fitted and included in the multivariable Cox proportional hazard regression model. In the multivariable analysis, an adjusted hazard ratio (AHR) with its 95% confidence interval (CI) and *p*-value ≤0.05 were used to determine the strength of the relationship and to identify statistically significant variables, respectively.

## Results

A total of 614 charts of adult patients with COVID-19 were reviewed, of which 602 (98.04%) records met the enrollment criteria in the final analysis. The remaining 12 charts were incomplete because the date of hospitalization or the final outcome (death or censored) was not recorded. Out of the 602 adult COVID-19 charts followed for 8 months, 514 were censored and 87 died.

### Sociodemographic characteristics of the study participants

Of the 602 study participants, 381 (63.29%) were men. Regarding the age distribution, the median age of the study participants was 41 years and the mean age was 44.8 ± 18.9 years.

### Clinical features of the study participants on admission

The majority of study participants (*n*=518, 86.05%) had a history of one or more symptoms at admission. Most of the participants (*n*=473, 78.57%) had a cough on admission, followed by fever (*n*=240, 39.87%), shortness of breath (*n*=239, 39.70%), fatigue 134 (22.26%), myalgia 87 (14.45%), chest pain 80 (13.29%), and sore throat (*n*=77, 12.79%). The mean diastolic blood pressure on admission was higher among those who died than among those who were censored (84.6 vs. 76.8 mmHg). Regarding oxygen saturation, the mean SpO_2_ (%) among those who died was significantly lower than those who were censored (86.1% vs. 92.2%). Regarding the severity of COVID-19 illness on admission, 84 (13.9%) of the participants were asymptomatic, 304 (64.4%) were mild, 64 (10.6%) were moderate, and 150 (24.9%) were severe.

### Baseline laboratory marker of the study participants

Concerning the baseline complete cell count, the mean WBC count of study participants was higher among those who died than those who were censored (13.7 vs. 7.33). The mean RBS of study participants who died and who were censored was 140 and 126.2 mg/dl, respectively. The majority of participants (*n*=252, 44.2%) were of the blood type “B,” followed by the blood group “A” (*n*=213, 37.3%) (Table 1).

**Table 1:** Baseline laboratory marker of adult COVID-19 patients at Eka Kotebe General Hospital, COVID-19 Treatment Center, Addis Ababa, Ethiopia, 2021 (*n* = 602).

Variables		Mean ± SD	Status	
			Death (mean)	Censored (mean)
Complete cell count	White blood cell (10^3^/µl)	8.2 ± 5.1	13.7	7.33
	Neutrophil (10^3^/µl)	6.35 ± 2.6	8.78	5.94
	Monocyte (10^3^/µl)	0.50 ± 0.3	0.45	0.51
	Lymphocyte (10^3^/µl)	2.6 ± 3.11	1.76	2.79
	Red blood cell (10^6^/µl	5.0 ± 4.09	4.51	5.13
	Hemoglobin (g/dl)	14.1 ± 2.9	13.0	14.3
	Hematocrit (%)	39.77 ± 9.1	35.87	40.3
	Platelet count (cells/L)	224.9 ± 95.1	184.9	231.7
Renal function test (*n* = 551)	Urea (mg/dl)	19.9 ± 8.3	22.2	19.4
	Creatinine (mg/dl)	1.07 ± 1.00	1.72	0.95
Liver function test (*N* = 551)	Aspartate transaminase (U/L)	40.20 ± 20.9	49.44	38.4
	Alanine transaminase (U/L)	42.4 ± 27.45	51.6	40.69
Random blood sugar (mg/dl) (*n* = 419)	126.18 ± 65.9	140.0	126.2
Variable	Category	Death (%)	Censored (%)	Total (%)
Blood group (*n* = 570)	A	27 (4.75%)	186 (32.6%)	213 (37.3)
	B	44 (7.72%)	208 (36.5%)	252 (44.2%)
	AB	7 (1.23%)	16 (2.81%)	23 (4.04%)
	O	9 (1.58%)	73 (12.81%)	82 (14.4%)

### Pre-existing comorbidity status of study participants

Nearly half of the study participants (*n*=292, 48.5%) had at least one comorbidity; of them, 56 (18.86%) patients had three or more comorbidities. Among the comorbidities, hypertension was the most common (28.24%), followed by diabetes mellitus (23.26%), cardiac disease (9.63%), and renal diseases (6.81%) (Table 2).

**Table 2:** Pre-existing comorbidity status of adult COVID-19 patients at Eka Kotebe General Hospital, COVID-19 Treatment Center, Addis Ababa, Ethiopia, 2021.

Variables	Category	Status		Total (%)
		Death (%)	Censored (%)	
Having existing comorbidity	No comorbidity	13(4.19%)	297(95.81%)	310(51.5%)
	Have at least one comorbidity	74 (25.34%)	218 (74.66%)	292 (48.5%)
Number of comorbidities	1–2	53 (21.99%)	188 (78.01%)	241 (81.14%)
	≥3	22 (39.29%)	34 (60.71%)	56 (18.86%)
Cardiac disease (CHF)	No	54(10.09%)	481(89.91%)	535(88.87%)
	Yes	33(49.25%)	34(50.75%)	58(9.63%)
Hypertension	No	45 (10.42%)	387 (89.58%)	432 (71.76%)
	Yes	42 (24.71%)	128 (75.29%)	170 (28.24%)
Diabetes mellitus	No	37 (8.01%)	425 (91.99%)	462 (76.74%)
	Yes	50 (37.71%)	90 (64.29%)	140 (23.26%)
Chronic lung diseases (Chronic obstructive pulmonary disease (COPD) and/or asthma)	No	84 (14.8%)	496 (85.525)	580 (96.35%)
	Yes	3 (13.64%)	19 (86.36%)	22 (3.65%)
HIV/AIDS	No	81 (14.01%)	497 (85.99%)	578 (96.01%)
	Yes	6 (25%)	18 (75%)	24 (3.99%)
Malignancy	No	84 (14.26%)	505 (85.74%)	589 (97.84%)
	Yes	3 (23.08%)	10 (76.92%)	13 (2.16%)
Chronic liver disease	No	80 (13.63%)	507 (86.37%)	587 (97.51%)
	Yes	7 (46.57%)	8 (53.33%)	15 (2.49%)
Renal diseases	No	65 (11.55%)	498 (88.45%)	563 (93.52%)
	Yes	22 (56.41%)	17 (43.59%)	41 (6.81%)
Chronic neurological diseases	No	79 (13.64%)	500 (86.36%)	579 (96.18%)
	Yes	8 (34.78%)	15 (65.22%)	23 (3.82%)

### Treatment-related characteristics of study participants

Nearly half (n=297, 49.34%) the study participants received azithromycin and 189 (31.4%) were given dexamethasone. In total, 122 (20.27%) and 75 (12.46%) participants received ceftriaxone and vancomycin treatment. Only 12 (1.99%) of the participant were given chloroquine.

### Survival status of patients with COVID-19

In the present study, 602 adult patients with COVID-19 were followed retrospectively. The median hospital stay was 13 days, with a minimum and maximum follow-up time of 2 and 58 days, respectively. In this study, 514 patients were censored and 87 died, resulting in a total cumulative incidence of death of 14.4% during the follow-up period. The total follow-up time was 8,109 person-days, with an incidence rate of 10.7 deaths per 1,000 person-day observations (95% CI 8.79–13.38).

### Overall survival rate of patients with COVID-19

**Figure 1:**
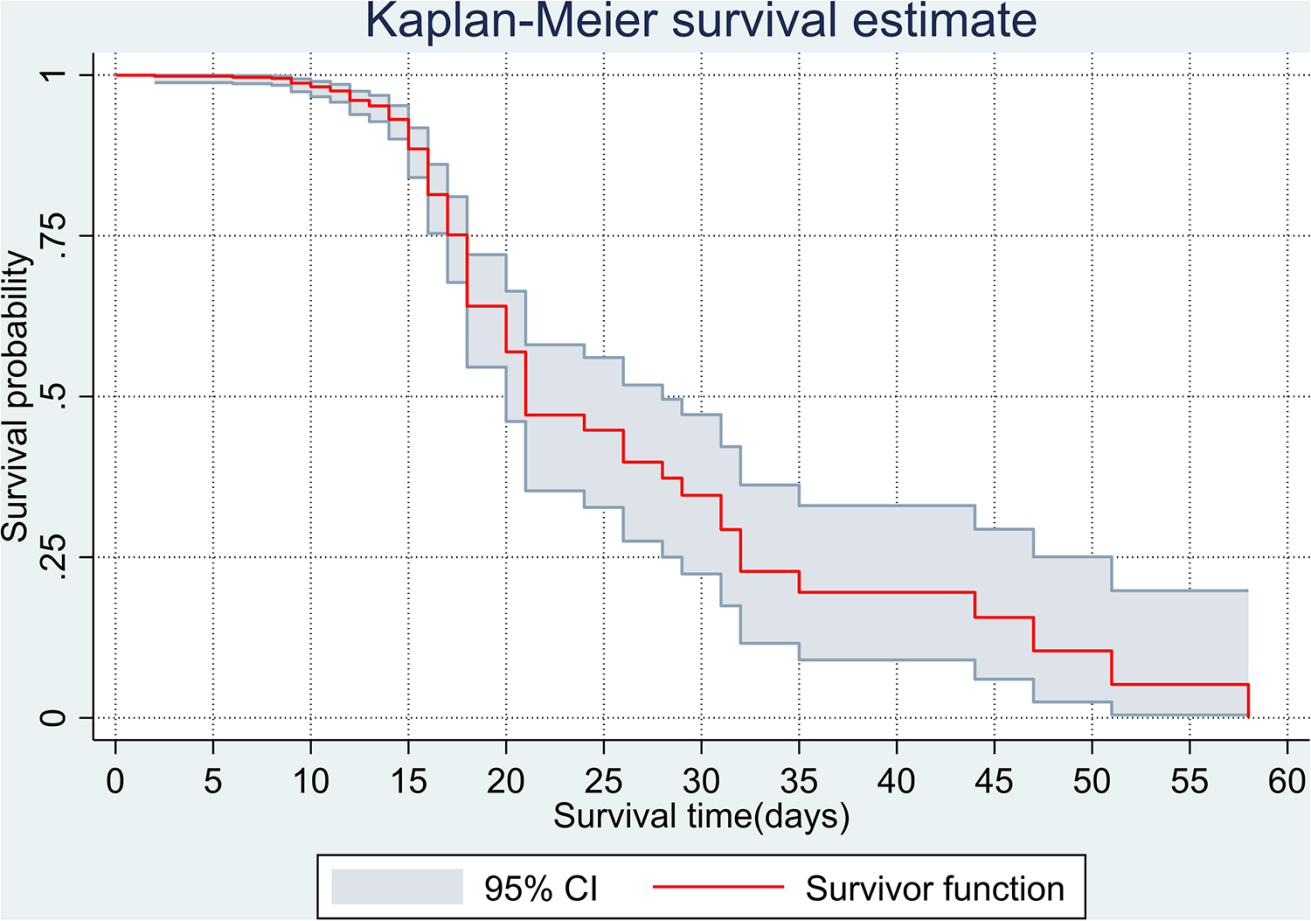
Overall Kaplan–Meier survival estimate of adult COVID-19 patients in Ethiopia, 2021.

**Figure 2:**
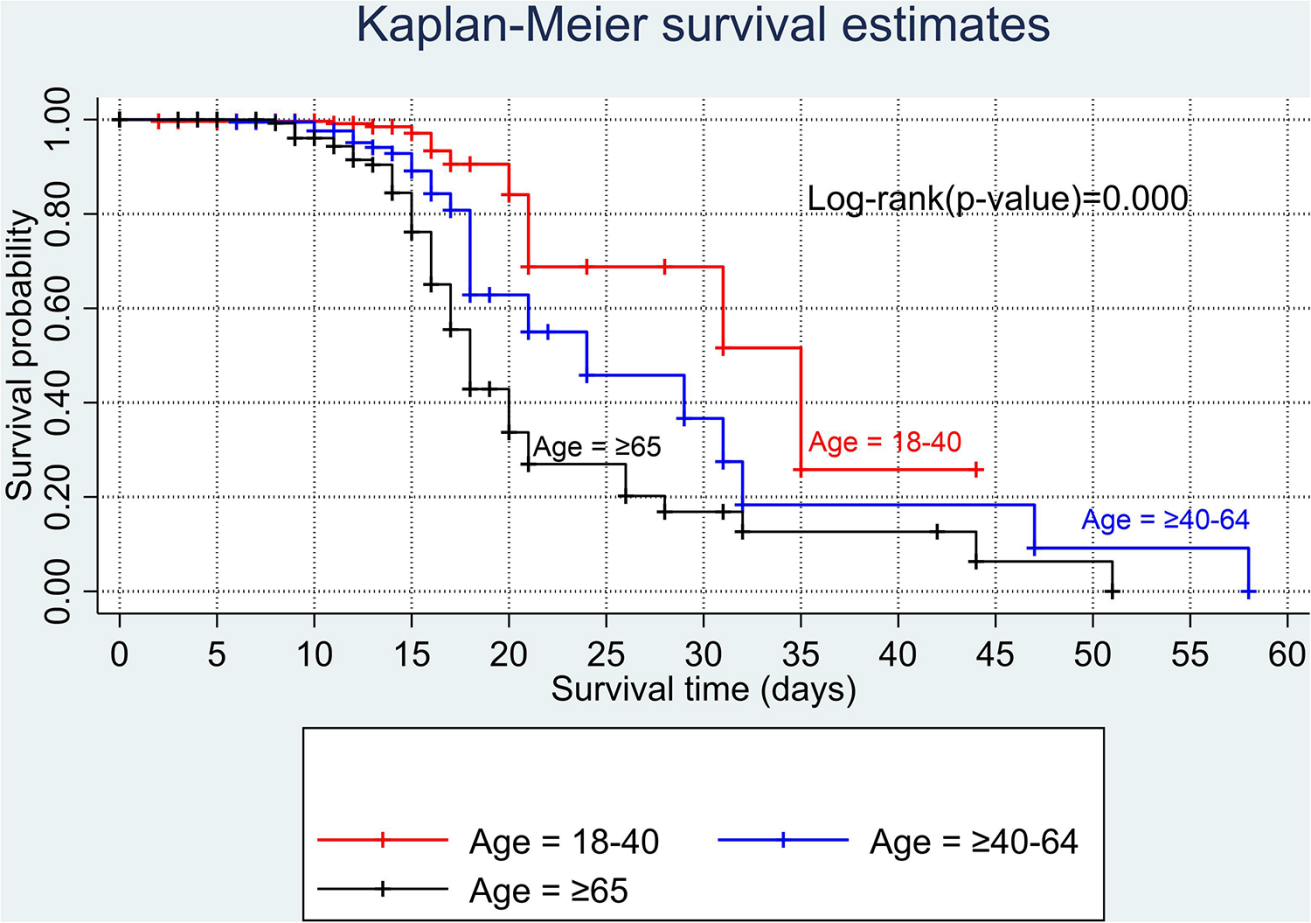
Kaplan–Meier survival curve by age categories among adult COVID-19 patients.

**Figure 3:**
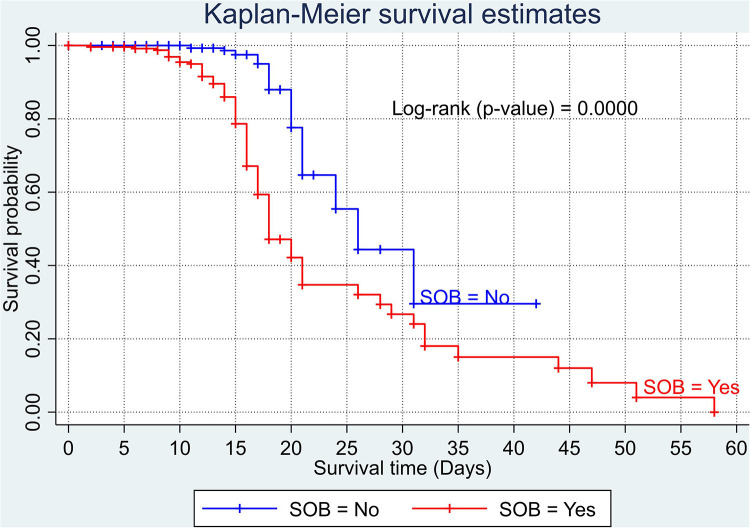
Kaplan–Meier survival curve based on the presence of shortness of breathing at admission among adult COVID-19 patients.

**Figure 4:**
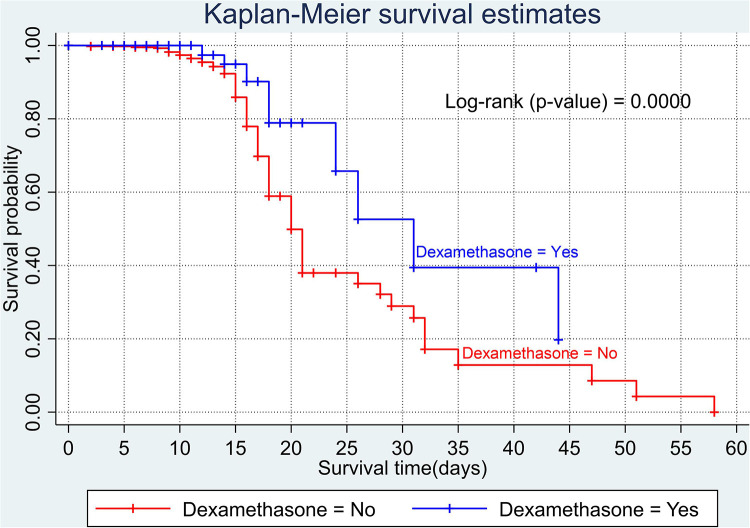
Kaplan–Meier survival curve by dexamethasone treatment among adult COVID-19 patients.

**Figure 5:**
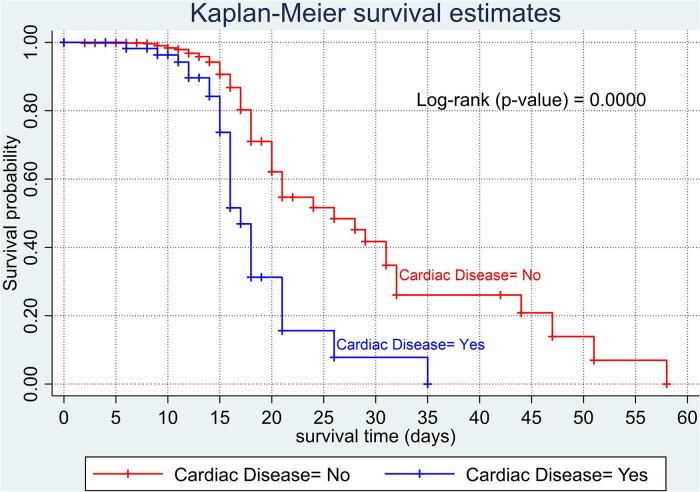
Kaplan–Meier survival curve based on the presence of cardiac disease (heart failure) among adult COVID-19 patients.

**Figure 6:**
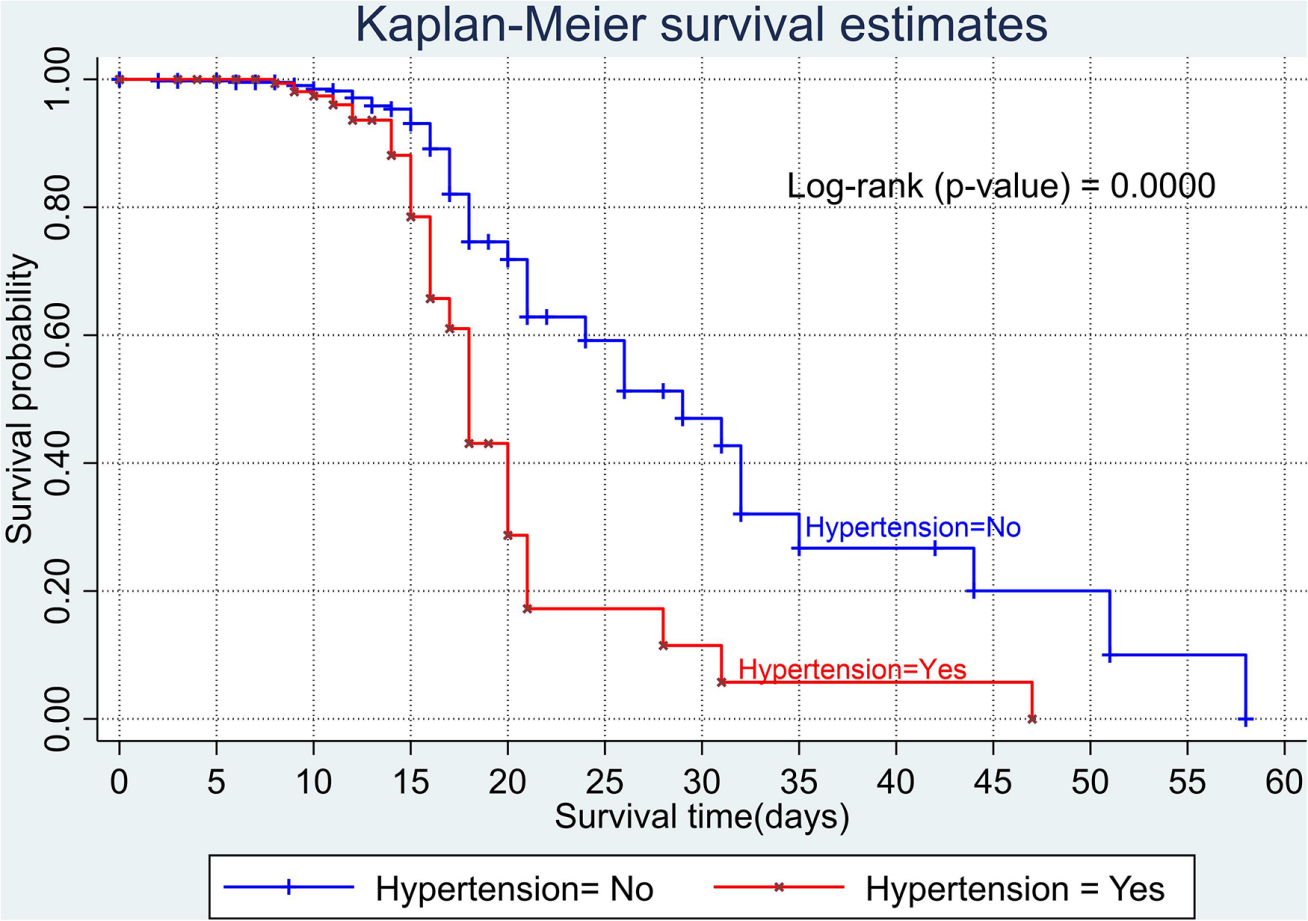
Kaplan–Meier survival curve based on the presence of hypertension among adult COVID-19 patients.

**Figure 7:**
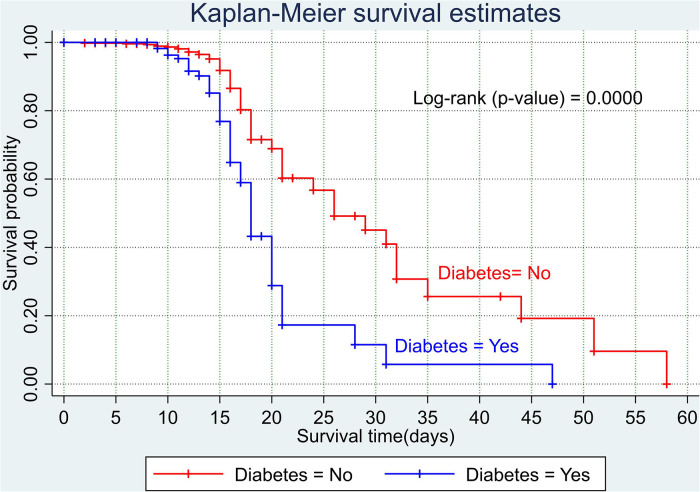
Kaplan–Meier survival curve based on the presence of diabetes among adult COVID-19 patients.

The overall Kaplan–Meier estimate showed that the probability of survival of patients with COVID-19 was high on the first day of admission and falls as the follow-up time increases. The overall median survival time of adult patients with COVID-19 was 21 days (Figure 1). The mean survival time of the study participants was 27.23 days (95% CI 23.82–30.63). The probability of survival for patients with COVID-19 at the start of follow-up was 100%. The probability of survival at 2, 3, 5, and 10 days was 99.8%, 99.5%, 97.6%, and 76.7%, respectively. The Kaplan–Meier curve with log-rank *p*-value shows differences in survival function between the categories of variables: age (Figure 2), shortness of breath at admission (Figure 3), dexamethasone treatment status (Figure 4), cardiac disease (Figure 5), hypertension (Figure 6), and diabetes (Figure 7).

### Predictors of COVID-19 mortality

In the multivariable Cox proportional hazards model, age, sex, shortness of breath, having existing comorbidities, diabetes mellitus, cardiac disease, and WBC (10^3^/µl) were significant predictors of COVID-19 mortality with a *p*-value of <0.05.

The multivariable analysis (Table 3) revealed that adults with COVID-19, who were aged 65 years or older, had a more than twofold risk of death (AHR 2.22, 95% CI 1.02–4.86) than those adults in the age group of 18–40 years. The risk of death from COVID-19 was nearly three times higher (AHR 3.04, 95% CI 1.61–5.74) for men than for women. Patients with a shortness of breath at admission had more than twice (AHR 2.29, 95% CI 1.16–4.54) the risk of death than those patients without shortness of breath at admission. Patients with COVID-19 with at least one comorbidity had a more than twofold risk of death (AHR 2.23, 95% CI 1.04–4.80) than those with no comorbidities. The risk of death from COVID-19 was more than twofold (AHR 2.31, 95% CI 1.30–4.08) for patients with diabetes than those without diabetes. Patients with cardiac disease had a more than twofold risk of death (AHR 2.07, 95% CI 1.21–3.43) from COVID-19 than those without cardiac disease. Furthermore, patients with a baseline WBC count greater than 10 (10^3^/µl) had a more than twofold risk of death (AHR 2.62, 95% CI 1.55–4.44) than those with a baseline WBC count within the normal range (≥4–≤10 [10^3^/µl]).

**Table 3:** Cox proportional hazard model analysis for predictors of time to death among adults with COVID-19 at Eka Kotebe General Hospital, COVID-19 Treatment Center, Addis Ababa, Ethiopia, 2021.

Independent variables	Category	Bivariable cHR (95% CI)	Multivariable AHR (95% CI)
Age	18–40	1	1
	≥40–64	2.63 (1.31–5.28)**	2.18 (0.92–5.18)
	≥65	4.96 (2.63–9.36)***	2.22 (1.02–4.86)*
Sex	Female	1	1
	Male	2.03 (1.22–3.38)**	3.04 (1.61–5.74)***
Fever	No	1	1
	Yes	1.32 (0.84–2.06)	0.60 (0.35–1.04)
Shortness of breathing	No	1	1
	Yes	4.58 (2.56–8.19)***	2.29 (1.16–4.54)*
Myalgia	No	1	1
	Yes	2.07 (1.27–3.38)**	0.94 (0.49–1.78)
Fatigue	No	1	1
	Yes	1.29 (0.84–1.99)	1.22 (0.68–2.19)
Having existing comorbidity	No	1	1
	≥1 comorbidity	4.90 (2.71,8.87)***	2.23 (1.04–4.80)*
Hypertension	No	1	1
	Yes	2.67 (1.74–4.12)*	0.73 (0.41–1.33)
Diabetes mellitus	No	1	1
	Yes	2.63 (1.70–4.06)***	2.31 (1.30–4.08)**
Cardiac disease (CHF)	No	1	1
	Yes	2.98(1.85–4.81)	2.07(1.21–3.43)**
Renal diseases	No	1	1
	Yes	3.25 (1.98–5.33)***	1.47 (0.82–2.63)
White blood cell (10^3^/µl)	<4	0.70 (0.32–1.59)	0.56 (0.23–1.48)
	≥4–≤10	1	1
	>10	4.19 (2.57–6.84)***	2.62 (1.55–4.44)***
Platelet count (cells/L)	≤150	1.68 (1.08–2.61)*	1.09 (0.67–1.80)
	>150	1	1
Dexamethasone	No	1	1
	Yes	0.48 (0.27–0.86)	0.54 (0.28–1.01)
Unfractionated heparin	No	1	1
	Yes	0.57 (0.32–1.01)	0.54 (0.27–1.09)

cHR, Crude hazard ratio; AHR, adjusted hazard ratio.

* Predictors at *P*-value < 0.05; **Predictors at *P*-value < 0.01; ***Predictors at *P*-value < 0.001.

## Discussion

The present study aimed to assess the survival status and predictors of mortality among adults with COVID-19 attending Eka Kotebe General Hospital, COVID-19 Treatment Center. This study shows that the overall cumulative mortality rate in an adult with COVID-19 during the study period was 14.4%. This finding is lower than the study carried out in DRC (29%) (32), China (28%) (33), New York City (43%) (34), and Brazil (46.25%) (35). The discrepancy of results that have been seen among studies might be due to the high prevalence of comorbidity recorded in studies from New York City (77%) and Brazil (70.9%) and the high number of older adults (aged ≥65 years) in the study from DRC compared to this study (31.9% vs. 19.93%, respectively) (36). Furthermore, this retrospective follow-up study includes patients with mild symptoms and a high number of younger adults than the above studies.

On the other hand, the overall cumulative mortality rate in the present study is higher than the studies carried out in Egypt (11.7%) (37) and Denmark (5.5%) (21). The difference is likely to be due to the fact that, later in our setup, health resources are restricted and admission becomes available for moderate and severe cases. This may increase the mortality rate in the hospital. The mortality rate in this study is consistent with the study in Italy (14.4%) (38).

The present study revealed that the probability of survival at 2, 3, 5, and 10 days was 99.8%, 99.5%, 97.6%, and 76.7%, respectively. The probability of survival in the present study was higher than in a study conducted in DRC (90%, 78.7%, 73.0%, and 71.6%, respectively) (39), in Mexico (86.9%) (40) at 3 days, and Brazil (79.21% and 59.22%) (41) at 5 and 10 days, respectively. In contrast, the findings of the present study were lower than the study conducted in another setting in Brazil, with a probability of survival of 95.1% at 10 days (42). This difference might be due to demographic characteristics. Furthermore, this discrepancy may be due to a lack of an early screening program and a higher proportion of severe COVID-19 at admission. It may also be due to variations in follow-up time, study period, and the clinical features of patients.

In this study, adults’ overall median survival time with COVID-19 was found to be 21 days. This finding is higher than the study carried out in DRC (9 days) (43), New York City (9 days) (14), Brazil (12 days) (26), and Fortaleza (Northeast Brazil) (19 days) (44). A possible explanation might be the high number of severely or critically ill patients. For example, the study conducted in DRC, at Kinshasa University Hospital, is in a tertiary care hospital. A high number of critically ill patients were referred from other hospitals. Therefore, patients may die within a short period of time.

In addition, a possible explanation for variations in the median survival time could be due to different follow-up times. In this study, there is a long follow-up time compared to the above studies. However, this finding was lower than that in the study carried out in Tongji Hospital, China (25 days) (45), and Germany (25 days) (26). This could be due to the difference in the quality of care in the cases of COVID-19 between low-income and high-income countries, such as a shortage of ventilator machines (46).

According to the results of this study, age was found to be a significant predictor of COVID-19 mortality. Older adults (aged ≥65 years) had an almost twofold risk of dying than young adults (aged 18–40 years). This finding points out that older people with early symptoms of COVID-19 should seek care immediately. This finding is supported by other previous studies conducted in African countries (47, 48), Denmark (49), China (50), and the United States and Mexico (42). A possible explanation might be that older adults were susceptible to multiple organ failure and comorbidities, increasing the likelihood of dying. Furthermore, a recent study revealed that older adults are at high risk of mortality with COVID-19 due to four main reasons: (1) the existence of asymptomatic systemic inflammation in the absence of significant disease; (2) a reduced immune system and type I interferon response due to long-lasting inflammation; (3) downregulation of ACE2 receptors; and (4) biological aging (26).

Sex is also found to be another critical predictor of COVID-19 mortality. Men have a nearly threefold higher risk of mortality compared to women. This finding is consistent with the studies carried out in China (44, 51, 52) and the United States (53–55). This may be due to recent emerged scientific evidence that COVID-19 in men can be worsened by androgen-enabled expression of ACE2 receptors, a permissive feature that engages the SARS-CoV-2 spike protein for infection (14). In addition, the staggeringly high mortality rate of COVID-19 in men may be partly explained by their pre-existing diseases (cardiac diseases, hypertension, and diabetes), higher risk behavioral patterns (cigarette smoking and alcohol consumption), and occupational exposure.

The risk of death among patients who presented with shortness of breath at admission was 2.29 times higher than those with no such symptom. Such a finding implies that the early identification of a patient with shortness of breath on admission may save patients' lives. This finding is supported by the study carried out in Ethiopia (14). Having a shortness of breath is one of the expressions of lung problems, ranging from reduced lung function up to a life-threatening condition. An insistent shortness of breath in patients might indicate structural and/or functional problems in the lung. This may increase the patients’ vulnerability to diseases such as COVID-19 and decrease the likelihood of survival with the stress leading to poor outcomes.

Patients with COVID-19 with at least one comorbid condition had twice the risk of death compared to patients without comorbidities. This finding is supported by other studies (17, 56). Patients with diabetes as a pre-existing comorbidity are twice as likely to die than those who do not have diabetes. This finding is pretty close to the studies conducted in Iran (57) and China (18). It is possible that since being diabetic is directly associated with low immune function, increasing the risk of bacterial and viral infection, including COVID-19, results in a poor outcome. This finding highlights the importance of timely monitoring and greater conservative management for those individuals with comorbidities. Furthermore, these findings point out that those vulnerable individuals should be in the front line in receiving the COVID-19 vaccine.

Likewise, several studies were conducted in different parts of the world: Brazil (24), China (58), Iran (59), and New York City (60). In this study, having heart disease is also another predictor of COVID-19 mortality. The presence of heart disease increases the risk of dying twofold. This might be because one of the most common immediate causes of death in patients with COVID-19 is respiratory failure (46). Having existing comorbidities such as cardiac disease might increase the incidence of respiratory failure and consequently lead to a poor prognosis. This finding implies that in the care of cardiac patients with COVID-19, special care should be incorporated to prevent further injury to the vital organs, thus improving their chance of survival.

Having a baseline WBC count of more than 10 (103/µl) had a risk of death 2.62 times higher compared to the normal range (≥4 to ≤10). This implies that an early increase in WBC count might be considered an indicator of a poor outcome. This finding argues that early screening of the WBC count on admission is essential to identify and provide special care for those patients, thereby reducing the likelihood of dying with COVID-19. This finding is in line with studies done in Hungary (61), China (62, 63), and Iran (19). The possible reason might be that an increase in the baseline WBC count is an early indicator of serious infection, increasing the risk of dying. However, the exact mechanism of how patients with a higher WBC level were at a high risk of death is unclear.

Since the data were derived retrospectively from medical reports of the patients, some critical variables such as laboratory markers (C-reactive protein level, albumin, cardiac markers dimer, IL-6, lactate dehydrogenase, and serum ferritin), which were strong predictors of COVID-19 mortality in other studies, were not or inadequately recorded and not included in the analysis. This led to difficulty in knowing the role of those predictors on survival time and was underestimated in predicting hospital death. The Eka Kotebe General Hospital COVID-19 Treatment Center is the first in Ethiopia and has better equipment and care. Patients were transferred late in their illness from other hospitals, and through these circumstances, the mortality rate might be overestimated.

## Conclusion

Great efforts have been made to overcome the seriousness of COVID-19 diseases. However, the mortality rate does not show a significant decline. The probability of survival of patients with COVID-19 in this study was relatively high. Male sex, older adult (aged ≥65 years), having shortness of breath at admission, having at least one comorbidity, diabetes mellitus, cardiac disease, and baseline WBC count were found to be independent predictors of COVID-19 mortality. To the best of our knowledge, this is the first all-inclusive study in Ethiopia that gives insight into the survival status of patients with COVID-19. We believe it provides the best locally available evidence toward the different characteristics or nature of COVID-19 disease, thereby contributing to the initial identification of patients with a poor outcome.

## Data Availability

The raw data supporting the conclusions of this article will be made available by the authors, without undue reservation.
