# Supplementary material for: Time to death and its predictors among adult patients with COVID-19: A retrospective cohort study in Ethiopia
Source: Front Epidemiol. 2023 Jan 17;2:1065184. doi: 10.3389/fepid.2022.1065184 (PMC10911043; doi:10.3389/fepid.2022.1065184)
Supplement: Supplementary file 1 [file Datasheet1.pdf]

VIF and tolerance test to check the existence of multicollinearity between covariates

Variable	VIF	1/VIF
comorbidity	2.33	0.430054
Age	1.81	0.552842
Hypertension	1.77	0.566258
Shortness_~g	1.74	0.573512
Diabetes	1.50	0.664984
Fatigue	1.43	0.700421
Fever	1.42	0.702772
Myalgia	1.28	0.778568
Cardiac_di~e	1.23	0.810470
Renal_dise~s	1.22	0.821011
White_bloo~l	1.18	0.849954
Dexamethas~e	1.15	0.867451
Platelet_c~t	1.08	0.922296
Sex	1.05	0.956913
Unfraction~n	1.04	0.957368
Mean VIF	1.42	

Variance inflation factor (VIF) and tolerance were computed to check the existence of multicollinearity prior to running the multivariable Cox Proportional hazard regression model. If VIF is above 4 or Tolerance is below 0.25 indicates that multicollinearity might exist. In this study, the maximum VIF was 2.33 for comorbidity status with mean VIF of 1.42 and the minimum tolerance value is 0.43. Thus, there is no multicollinearity between covariates.
